# Epileptic spasms in infants: can video-EEG reveal the disease’s etiology? A retrospective study and literature review

**DOI:** 10.3389/fneur.2023.1204844

**Published:** 2023-06-09

**Authors:** Raffaele Falsaperla, Sarah Sciuto, Grete Francesca Privitera, Lucia Giovanna Tardino, Giuseppe Costanza, Alessandra Di Nora, Roberto Horacio Caraballo, Martino Ruggieri

**Affiliations:** ^1^Unit of Pediatrics and Pediatric Emergency, University Hospital Policlinico “Rodolico-San Marco”, Catania, Italy; ^2^Unit of Neonatal Intensive Care and Neonatology, University Hospital Policlinico “Rodolico-San Marco”, Catania, Italy; ^3^Pediatrics Postgraduate Residency Program, Section of Pediatrics and Child Neuropsychiatry, Department of Clinical and Experimental Medicine, University of Catania, Catania, Italy; ^4^Department of Clinical and Experimental Medicine, Department of Mathematics and Computer Science, University of Catania, Catania, Italy; ^5^Department of Neurology, Hospital de Pediatría “Juan P. Garrahan”, Buenos Aires, Argentina; ^6^Unit of Rare Diseases of the Nervous System in Childhood, Department of Clinical and Experimental Medicine, Section of Pediatrics and Child Neuropsychiatry, University of Catania, Catania, Italy

**Keywords:** epileptic spasms, video-EEG, electro-clinical pattern, newborn, seizure

## Abstract

**Objective:**

Epileptic spasms are a type of seizure defined as a sudden flexion or extension predominantly of axial and/or truncal limb muscles that occur with a noticeable periodicity. Routine electroencephalogram supports the diagnosis of epileptic spasms, which can occur due to different causes. The present study aimed to evaluate a possible association between the electro-clinical pattern and the underlying etiology of epileptic spasms in infants.

**Materials and methods:**

We retrospectively reviewed the clinical and video-EEG data on 104 patients (aged from 1 to 22  months), admitted to our tertiary hospital in Catania and the tertiary hospital in Buenos Aires, from January 2013 to December 2020, with a confirmed diagnosis of epileptic spasms. We divided the patient sample into structural, genetic, infectious, metabolic, immune, and unknown, based on etiology. Fleiss’ kappa (К) was used to assess agreement among raters in the electroencephalographic interpretation of hypsarrhythmia. A multivariate and bivariate analysis was conducted to understand the role of the different video-EEG variables on the etiology of epileptic spasms. Furthermore, decision trees were constructed for the classification of variables.

**Results:**

The results showed a statistically significant correlation between epileptic spasms semiology and etiology: flexor spasms were associated with spasms due to genetic cause (87.5%; OR < 1); whereas mixed spasms were associated with spasms from a structural cause (40%; OR < 1). The results showed a relationship between ictal and interictal EEG and epileptic spasms etiology: 73% of patients with slow waves and sharp waves or slow waves on the ictal EEG, and asymmetric hypsarrhythmia or hemi hypsarrhythmia on the interictal EEG, had spasms with structural etiology, whereas 69% of patients with genetic etiology presented typical interictal hypsarrhythmia with high-amplitude polymorphic delta with multifocal spike or modified hypsarrhythmia on interictal EEG and slow waves on the ictal EEG.

**Conclusion:**

This study confirms that video-EEG is a key element for the diagnosis of epileptic spasms, also playing an important role in the clinical practice to determine the etiology.

## 1. Introduction

Epileptic spasms (ES) are a type of seizure defined as a sudden flexion, extension, or mixed extension–flexion of predominantly proximal and truncal muscles. Usually, they are more sustained than a myoclonic movement but not sustained as a tonic seizure (1). ES have been described as grimacing, head nodding, or subtle eye movements and frequently occur in clusters. The diagnosis of infantile ES is made by a combination of the typical clinical features with a electroencephalogram (EEG) or electromyography (EMG) (2).

Studies of electroclinical manifestations of infantile spasms using video EEG are rare in the literature (3–7). Commonly, the EEG pattern in epileptic spasms shows a multiphasic slow wave transient, which is diffuse but maximal in frontal derivations on EEG, followed by a second fast rhythm or brief polyspike burst of low amplitude, predominantly in the central regions (8–11). During the tonic phase of the seizure, the EEG shows an attenuation with superimposed fast activity.

The West Delphi Group (2) recommends a degree of standardization in the timing of EEG investigations; video-EEGs have shown increased sensitivity with longer recording periods and the inclusion of non-REM sleep (2, 12). Taken together, the findings from the EEG-video study allow an adequate diagnosis and classification of the different epileptic seizures and syndromes, which can optimize both the aetiological investigation and therapeutic management (13, 14). It could be very helpful to make recordings using a polygraphic channel, as this may show the characteristic diamond-shaped EMG correlated to the spasms (15). Once the diagnosis is established, efforts should be made to find the underlying etiology, as this significantly affects treatment decisions and prognosis. Some years ago, the etiology of epileptic spasms was largely unknown (8). In the literature, studies suggest that epileptic spasms are not etiologically specific, but rather the result of a variety of different insults (11). Actually, numerous series of causes are recognized as follows: structural, genetic, metabolic, or acquired. Understanding the underlying causes has an important role for clinicians. Magnetic resonance imaging is important to identify the etiology of the disease (16). Although some causes have been brought to light in recent years, more studies need to elucidate the causes and find diagnostic tools (17).

## 2. Purpose

The aim of the study was to analyze the electro-clinical pattern in infantile epileptic spasms and correlate it with the underlying etiology.

## 3. Materials and methods

### 3.1. Subjects

We retrospectively reviewed the medical records and the video-EEG monitoring results of 104 infants suffering from infantile spasms, all confirmed by polygraphy. The patients were admitted to the University Hospital “Policlinico-San Marco” in Catania and to the University Hospital de Pediatría “Prof. Dr. Juan P, Garrahan” in Buenos Aires, Argentina, from January 2013 to December 2020. The study was approved by the ethics committees of both hospitals.

We collected the data from the medical charts: sex, age, etiology, electroencephalogram (EEG), and neurological evaluation. For the study, we grouped children according to the causes of ILAE classification of the epilepsies (1) into six groups: structural, genetic, infectious, metabolic, immune, and unknown.

The structural causes consist of hypoxic–ischemic encephalopathy (HIE), malformations shown in the MRI, porencephaly, cortical dysplasia, brain tumor, intracranial hemorrhage, and stroke. The genetic causes, however, included tuberous sclerosis, Down syndrome, 1p36 deletion, and mutation of the SCN1A, ARX, and CDKL5 genes. The term unknown was used to describe patients with “unidentified” causes and normal brain MRIs. We included children fulfilling the Consensus Statement of the West Delphi Group (2) criteria for infantile spasms, with an age at onset between 1 and 24 months (Table 1).

**Table 1:** Inclusion and exclusion criteria.

Inclusion criteria	Exclusion criteria
Age at onset <24 months	Age at onset >24 months
Spasms occur in cluster	Infantile spasms single-spasm variant
Hypsarrhythmia (or modified hypsarrhythmia)	EEG without hypsarrhythmia
EEG before drug therapy	EEG after drug therapy

Polygraphic video-EEG findings were analyzed independently by three investigators, by reviewing interictal abnormalities while awake and asleep and the ictal EEG. We described the EEG paroxysms, spikes, and sharp waves, focusing on symmetry, voltage on spikes, and duration.

Inclusion and exclusion criteria were defined as shown in Table 1.

### 3.2. Video EEG

A prolonged EEG with synchronized video (video-EEG) improves the sensitivity, specificity, and diagnostic yield by attempting to record the habitual events when they are frequent (18, 19). In this study, we used head caps with prewired electrodes located according to the full 10–20 pad placement system. In 1-month-old babies and when the size of the head was too small, we used cup-shaped silver electrodes that were kept adherent to the skin using conductive and adhesive pastes and positioned according to the restricted 10–20 pad placement system (20). Both awake and sleep EEGs were obtained in all our cases, with recordings with a minimum duration of 150 min for each patient. Electrodes and movement artifacts were the most encountered because of the muscle movements during the ictus. Three authors (R.F., L.T., and R.H.C) independently evaluated each video-EEG. Fleiss’ kappa (К) (21) was used to assess agreement among raters in the electroencephalographic interpretation of hypsarrhythmia. An online calculator (dfreelon.org) was used to calculate К. Landis and Koch (22) interpreted the К as follows: <0: low agreement, 0.01–0.2: slight agreement, 0.21–0.4: fair agreement, 0.41–0.6: moderate agreement, 0.61–0.8: substantial agreement, and 0.81–1: nearly perfect agreement.

The ictal EEG correlates of the spasms were classified on the basis of voltage spikes background (mV), symmetric ictal activity, duration of spasms, and morphology (slow waves and sharp waves, fast rhythm, slow waves, high voltage slow waves followed attenuation, fast rhythms, and burst suppression). Interictal EEG (2) correlates were classified as interictal hypsarrhythmia typical with high amplitude polymorphic delta with multifocal spike, modified hypsarrhythmia, asymmetric hypsarrhythmia, and hemi hypsarrhythmia.

### 3.3. Statistical analysis

All analyzes were conducted using R(v.4.2.1). Logistic regression “glm” was computed through the caret package (v.6.0–93) (23) to understand the role of the different video-EEG variables on the etiology of ES with a multivariate analysis and with a bivariate analysis. Additionally, Fisher’s exact test was used to compare each variable across the major possible etiologies. The results were considered significant with value of *p* <0.05. The odds ratio (OR) was calculated for each analysis. Furthermore, recursive PARTitioning (rpart) *via* the rpart(v.4.1.16) and rpart.plot (v.3.1.1) packages was employed to build decision trees for variable classification (24).

## 4. Results

**Figure 1:**
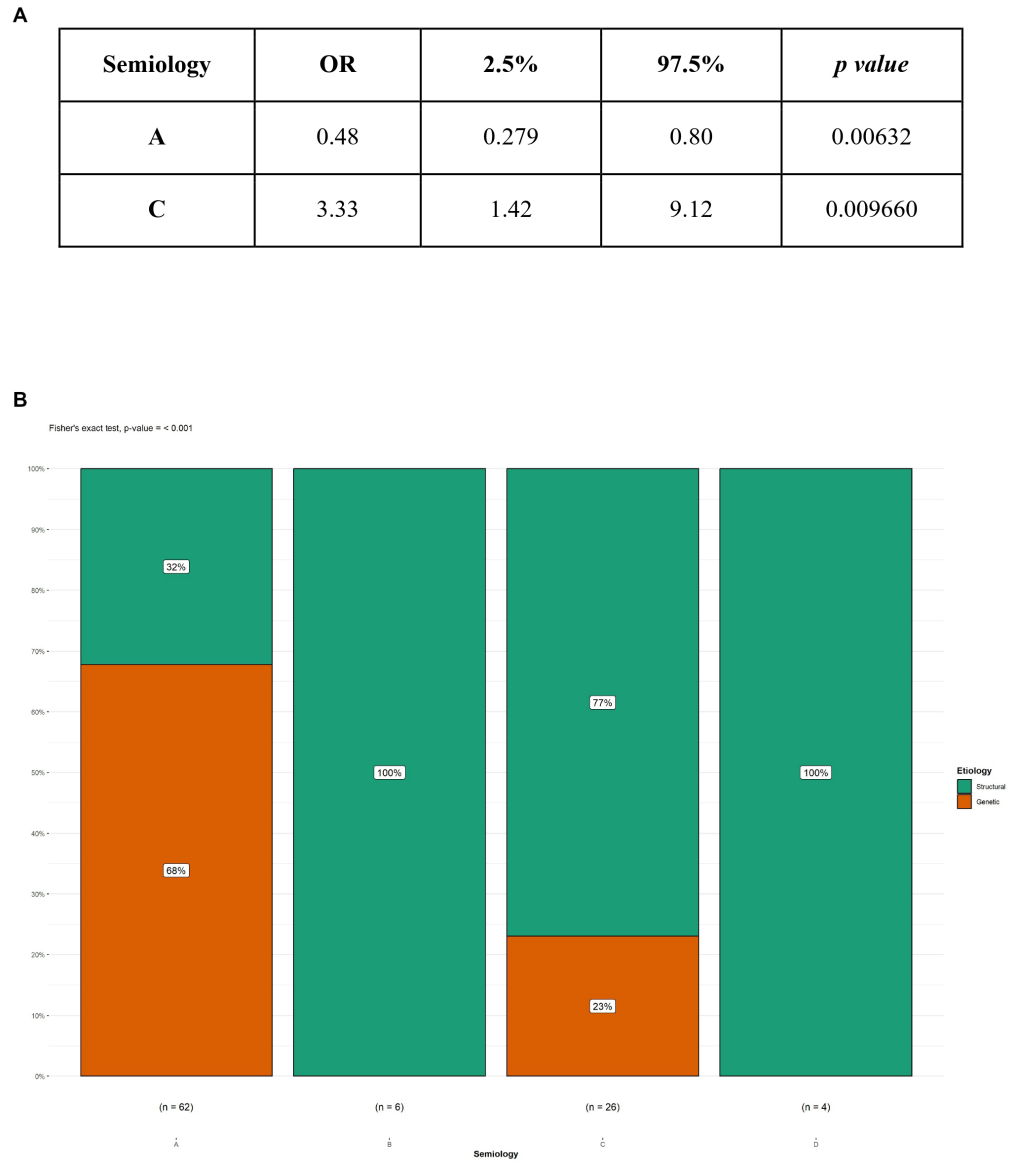
Logistic regression and Fisher’s test between etiology and type A and C semiology. **(A)** Logistic regression; **(B)** Fisher’s test; OR, Odds ratio; A, flexor spasms; C, mixed spasms.

**Figure 2:**
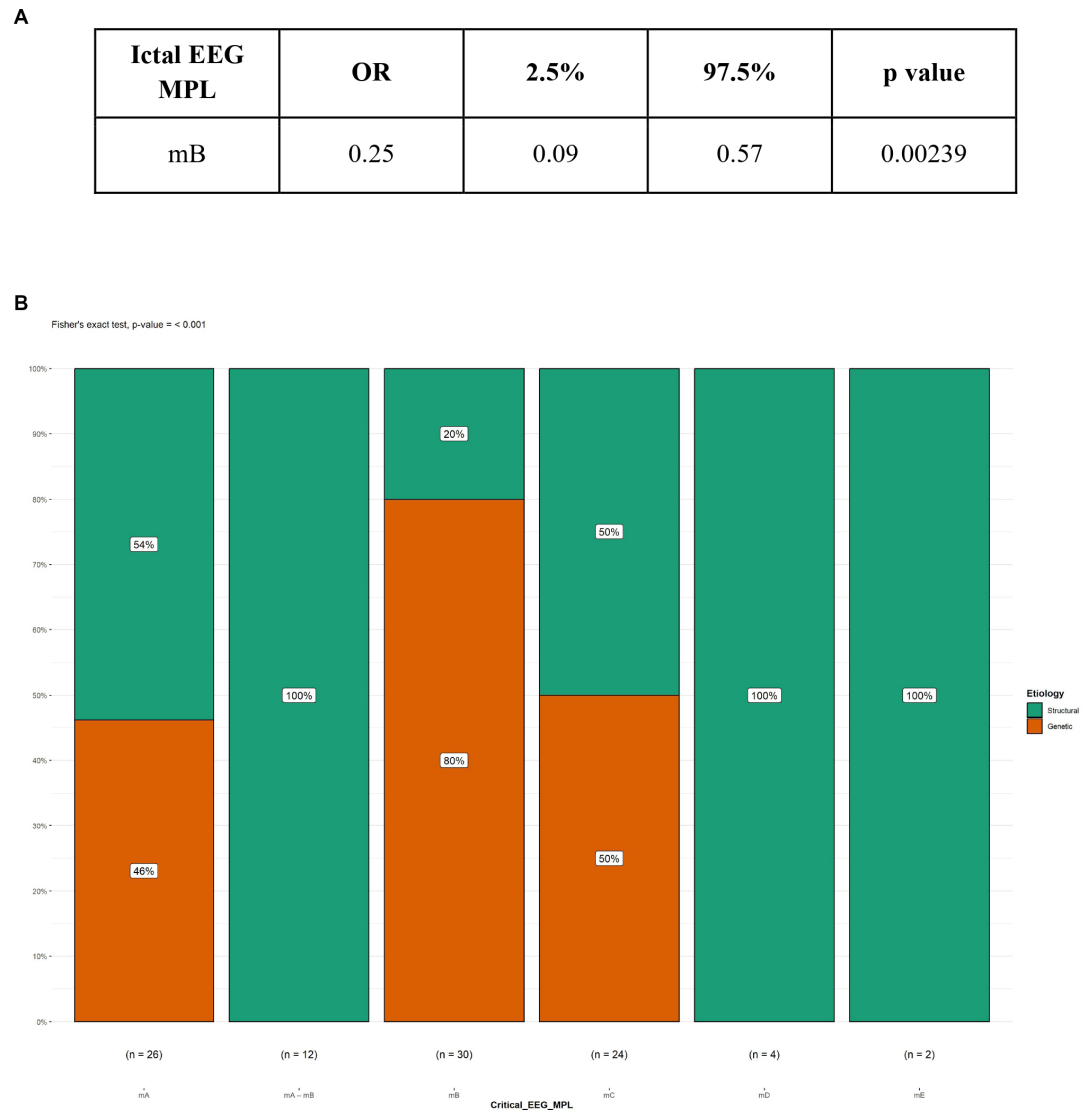
Logistic regression and Fisher’s test between Etiology and ictal EEG morphology. **(A)** Logistic regression; **(B)** Fisher’s test; MPL, morphology; mB, fast rhythm; OR, Odds ratio.

**Figure 3:**
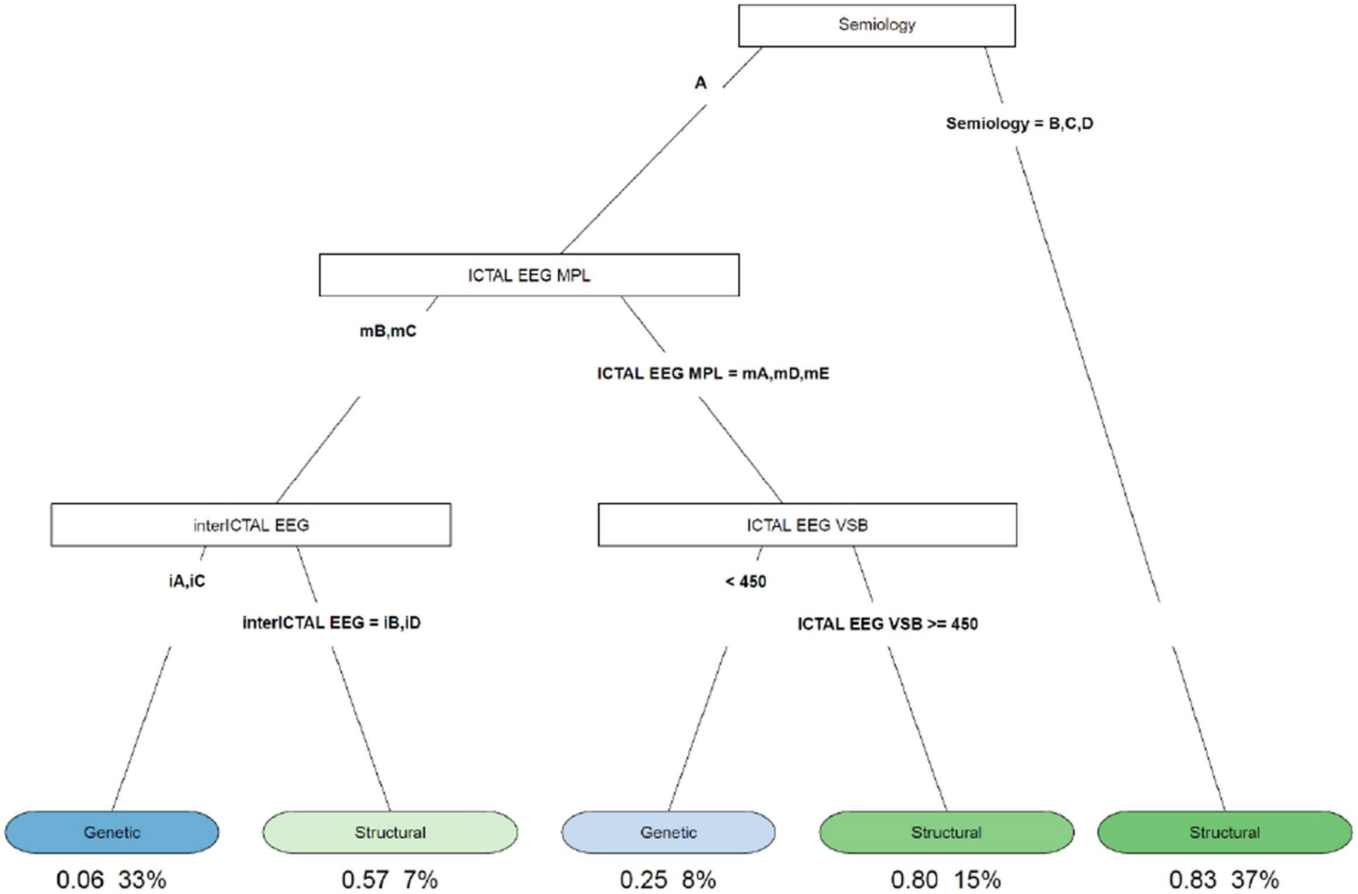
Decision tree of the correlation between type of spasm, EEG characteristics (ictal and interictal EEG), and etiology. A, flexor spasms; B, extensor spasms; C, mixed spasms; D, focal seizures followed by spasms; iA, interictal hypsarhythmia typical with high amplitude polymorphic delta with multifocal spike; iB, modified hypsarhythmia; iC, asymmetric hypsarhythmia; iD, hypsarhythmia; mA, slow waves and sharp waves; mB, fast rhythm; mC, slow waves; mD, high voltage slow waves followed attenuation; mE, fast rhythm and burst suppression; VSB, voltage spikes background; MPL morphology.

**Figure 4:**
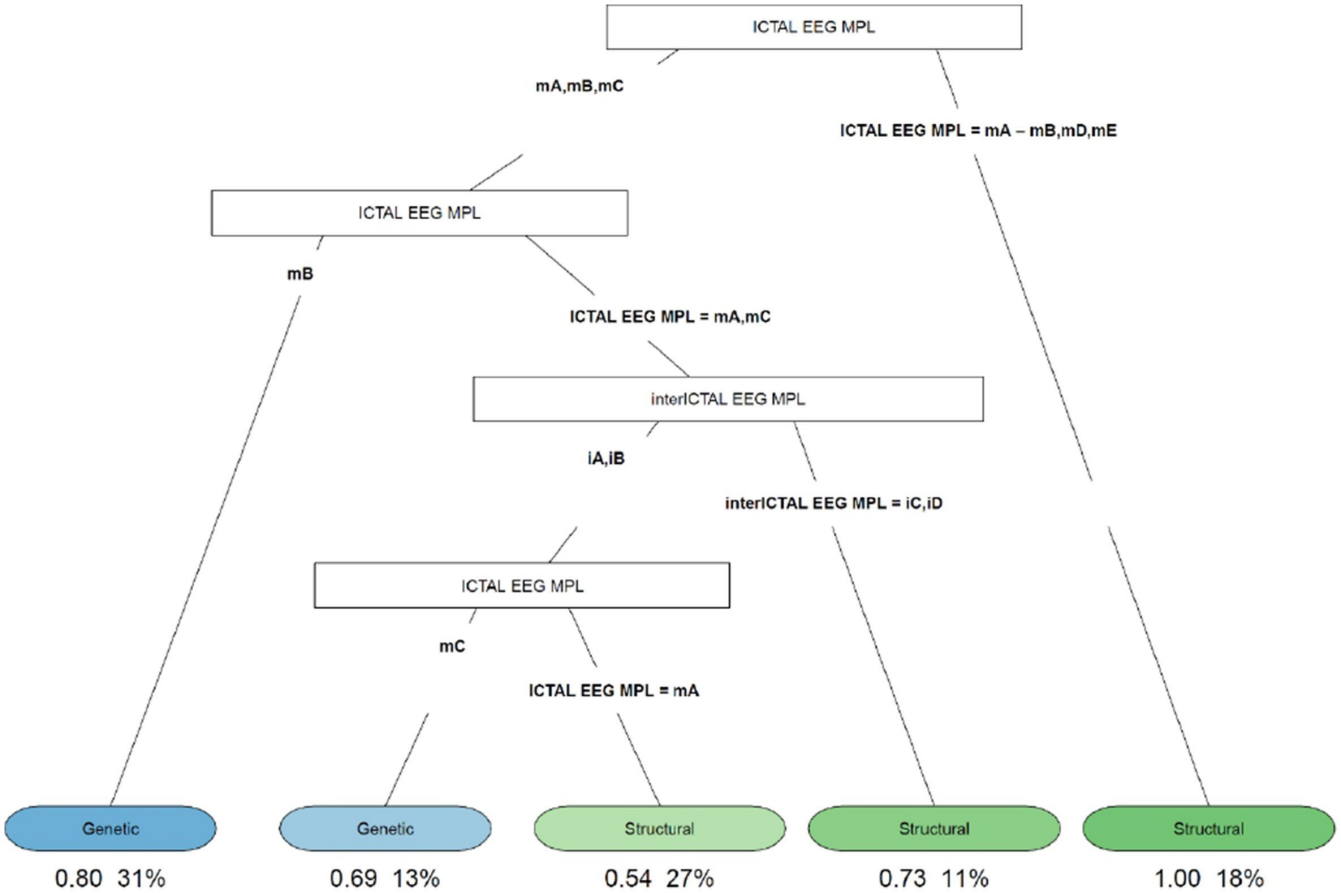
Decision tree of the correlation between EEG characteristics (ictal and interictal EEG) and etiology. iA, interictal hypsarhythmia typical with high amplitude polymorphic delta with multifocal spike; iB, modified hypsarhythmia; iC, asymmetric hypsarhythmia; iD, hypsarhythmia; mA, slow waves and sharp waves; mB, fast rhythm; mC, slow waves; mD, high voltage slow waves followed attenuation; mE, fast rhythm and burst suppression; VSB, voltage spikes background; MPL morphology.

In our study, 104 patients affected by infantile spasms, 56 (53,8%) female patients and 48 (46,2%) male patients, were retrospectively analyzed. The age at diagnosis ranged from 1 month to 22 months with a mean age of 7.5 (± 4.12 months). The results obtained were grouped based on the etiology of the epileptic spasms: structural (50 patients, 48%), genetic (48 patients, 46%), unknown (5 patients, 5%), infectious (1 patient, 1%), metabolic (0 patients), and immune (0 patients). Because of the small number of patients in most categories, we chose to report on only the most represented etiologies: structural and genetic (Table 2). Table 3 illustrates the baseline and clinical characteristics, as well as the details of the interictal and ictal EEG, of all patients with structural and genetic spasms. Logistic regression between the etiology and semiology of spasms (Figure 1A) and between etiology and ictal EEG morphology (Figure 2A), indicated that flexor and mixed spasms and fast pace on ictal EEG were statistically significant compared to structural or genetic etiology. We realized two decision trees that described the correlation between the type of spasm, EEG characteristics, and etiology of the ES (Figure 3) and between the characteristics of the ictal and interictal EEG tracing with the etiology (Figure 4), respectively.

**Table 2:** Types of structural and genetic etiology.

Etiology	Patients (%)
Genetic	
TSC2	35.3
Trisomy 21	23.6
SCN1A	11.7
ARX	11.7
TSC1	5.9
CDKL5	5.9
Deletion 1p36	5.9
Structural	
Porencephaly	8.7
Cortical dysplasia	13
Heterotopy	4.3
Hemimegalencephaly	4.3
Hypoxic ischemic encephalopathy	17.5
Lissencephaly	8.7
Stroke	13
Intracranial hemorrhage	17.5
Birth trauma	13

**Table 3:** Details of epileptic spasms in patients with structural and genetic etiology.

Characteristics	Number of patients		*p* value
	Structural etiology	Genetic etiology	
Male/Female	23/27	12/36	<0.05 (0.0363)
Mean Age (months)	7.7 (± 4.9)	5.75 (± 2.95)	Ns
Psycomotor delay	44 (88%)	48 (100%)	<0.05 (0.0268)
Semiology			
A	20 (40%)	42 (87.5%)	<0.001
B	6 (12%)	0	<0.05 (0.0268)
C	20 (40%)	6 (12.5%)	<0.01 (0.0027)
D	4 (8%)	0	Ns
Symmetry	24 (48%)	12 (25%)	<0.05 (0.0221)
Average duration of cluster (min)	5.08 (± 3.6)	4.75 (± 3.6)	Ns
Interictal EEG			
iA	26 (52%)	36 (75%)	< 0.05 (0.0221)
iB	6 (12%)	6 (12.5%)	Ns
iC	16 (32%)	6 (12.5%)	< 0.05 (0.0288)
iD	2 (4%)	0	Ns
Ictal EEG			
VSB (mV)	487.5 (±120.4)	606 (±180.9)	< 0.001
SIA	24 (50%)	22 (44%)	Ns
DS (sec)	1.81 (±0.65)	1.61 (±0.7)	Ns
MPL mA	12 (25%)	14 (28%)	Ns
MPL mB	24 (50%)	6 (12%)	<0.001
MPL mA–mB	0	12 (24%)	<0.001
MPL mC	12 (25%)	12 (24%)	<0.001
MPL mD	0	4 (8%)	Ns
MPL mE	0	2 (4%)	Ns

A, flexor spasms; C, mixed spasms; iA, interictal hypsarhythmia typical with high amplitude polymorphic delta with multifocal spike; iB, modified hypsarhythmia; iC, asymmetric hypsarhythmia; mA, slow waves and sharp waves; mB, fast rhythm; mC, slow waves; VSB, voltage spikes background; SIA, symmetric ictal activity; DS, duration of spasms; MPL, morphology; Ns, no significant.

The decision tree in Figure 3 showed that 83% of the patients with extensor spasms, mixed spasms, and focal seizures followed by spasms were associated with spasms from a structural cause; patients with flexor spasms, fast rhythm or slow waves on the critical EEG, interictal hypsarrhythmia typical with high amplitude polymorphic delta with multifocal spike, and asymmetric hypsarrhythmia on all interictal EEG were 33% and of these, 6% had spasms due to genetic causes; patients with flexor spasms, with the presence of fast rhythm or slow waves on the ictal EEG, and with modified hypsarrhythmia or hemi hypsarrhythmia on the interictal EEG were 7% and of these, 57% had structural ES.

The decision tree in Figure 4 showed that if the ictal EEG presented slow waves and sharp waves (fast rhythms, high voltage slow waves followed by attenuation, or fast rhythms and burst suppression), all patients with this condition (18%) had ES due to structural causes. In cases of slow and sharp waves, fast rhythm, or slow waves showing up on the ictal EEG, we analyzed the cases as follows: (1) if a fast rhythm was present, in 80% of cases, it was ES from a genetic cause; (2) if a pattern with slow and sharp waves or slow waves was present, to distinguish between genetic and structural etiologies, the interictal EEG must be evaluated. If asymmetric hypsarrhythmia or hemi hypsarrhythmia was present on the interictal EEG, in 73% of cases these were spasms with structural etiology. If the interictal EEG showed typical interictal hypsarrhythmia with high-amplitude polymorphic delta with multifocal spike or modified hypsarrhythmia and slow waves on the ictal EEG, genetic etiology was found in 69% of cases; and (3) if slow and sharp waves were associated with the ictal EEG, in 54% of cases, the etiology was structural.

## 5. Discussion

In our study, we analyzed the electroclinical pattern in infantile epileptic spasms. In agreement with what Kannan and Katyayan affirmed in their studies (18, 19), we performed prolonged video EEG monitoring (minimum 150 min for each patient) in order to obtain good sensitivity, specificity, and diagnostic yield. Reviewers had near-perfect inter-rater agreement using the traditional method of EEG analysis to interpret hypsarrhythmia (К: 0.93).

Infantile spasms (IS) have been known to be associated with a wide variety of underlying pathologies. The most common causes of infantile spasms, according to the United Kingdom Infantile Spasms Study (UKISS), are hypoxic–ischemic encephalopathy (10%), chromosomal anomalies (8%), malformation (8%), perinatal stroke (8%), tuberous sclerosis complex (7%), and periventricular leukomalacia or hemorrhage (5%) (25). The etiologies we found were structural (48%), genetic (46%), unknown (5%), and infectious (1%). In our study, the greater incidence was found in female patients (64%), but in the literature, insignificant differences between the two sexes are described (26). The mean age for the entire sample is 7.25 months and there was no significant difference between etiological groups and age. Most of the children (94.2%) were symptomatic, in line with observations made in previous studies (27, 28). Psychomotor delay was present in almost all of the groups, therefore, it could not be considered a specific clinical sign.

Regarding the semiology of spasms, we compared the characteristics of spasms from structural causes with those from genetic causes. In particular, in genetic causes, flexor spasms were the most frequent (87.5%); in patients with structural causes, however, there was an equal percentage of flexor and mixed spasms (40%). Performing logistic regression (Figure 1A), flexor spasms and mixed spasms were found to be statistically significant in terms of etiology. In particular, flexor spasms (type A) were associated with spasms due to genetic causes; mixed spasms (type C), on the other hand, were associated with spasms from a structural cause. The result was confirmed by Fisher’s test (Figure 1B). In our study, more spasms that were asymmetrical were observed. Fusco and Iype (3, 27) noted that more than one child had spasms of various types and that symmetric spasms were present in the unknown etiology group and in the symptomatic patients group; in contrast, asymmetric spasms, or focal signs recognizable during a spasm, strongly indicated the existence of a cerebral lesion.

Cluster duration compared to the diagnosis showed no significant differences. The interictal EEG showed overall a predominance of the hypsarrhythmia typical with high amplitude polymorphic delta with multifocal spike form. Spike voltage compared to EEG diagnosis showed a significant difference between the structural and genetic forms (value of *p* <0.001). However, there was no significant difference regarding the symmetry of the ictal activity in the ictal EEG between the different diagnoses. In the sample we examined, among the patients suffering from structural forms of infantile spasms, the most frequent EEG morphology encountered was the slow and sharp waves (28%). In the cases with genetic etiology, the most recurring morphology was the fast rhythms. Logistic regression indicated that the presence of a fast rhythm on the ictal EEG was significantly associated with the presence of genetic spasms, with an OR of 0.25 (Figure 2A). The result was confirmed by Fisher’s test (Figure 2B).

There are varying patterns of ictal activity described in various studies (3–6). Kellaway et al. (5) noted that the ictal EEG pattern in infantile spasms varied from patient to patient and described 11 different types of ictal EEG patterns consisting of various combinations of generalized sharp or slow wave discharges, generalized voltage attenuation (electrodecremental discharges), and fast activity. They reviewed 5,042 spasms in 24 infants and noted that the generalized slow waves pattern followed or not by a period of voltage attenuation was present in 48.8% of seizures and a generalized sharp and slow waves complex followed or not by voltage attenuation was seen in 30.6% (total, 79.4%). Pachatz et al. (29), recorded diffuse slow waves in all 13 of their ictal records; fast activity was associated in 9/13 and associated voltage attenuation in one. In 1995, Haga et al. (4) noted high-voltage slow waves as ictal EEG correlation in all patients. In their study, the superposition of fast activity on slow waves was noted in the majority, and spike and slow wave activity in the minority.

The decision tree in Figure 3 showed that 83% of patients with extensor spasms, mixed spasms, and focal seizures followed by spasms were associated with spasms from a structural cause and that 57% of patients with flexor spasms, with the presence of fast rhythm or slow waves on the ictal EEG and with modified hypsarrhythmia or hemi hypsarrhythmia on the interictal EEG had structural ES.

Finally, the decision tree in Figure 4 found that 73% of patients with slow and sharp waves or slow waves on the ictal EEG, and asymmetric hypsarrhythmia or hemi hypsarrhythmia on the interictal EEG, had spasms with structural etiology. However, in 69% of patients with slow waves on the ictal EEG, and typical interictal hypsarrhythmia with high-amplitude polymorphic delta with multifocal spike or modified hypsarrhythmia on the interictal EEG, we found a genetic cause.

In the series by Fusco (3), in that by Haga (4), and by Iype (27), the ictal pattern did not correlate with the etiology as in our observation. Not even Pachatz et al. (29) were able to correlate the different spasm semiologies they recorded with the etiology and concluded that the occurrence of focal seizures with infantile spasms may be related to various etiologies.

## 6. Conclusion

The most frequent cause in our sample was represented by structural etiology followed by genetic etiology. Psychomotor retardation was not indicative and specific of a given etiology. There was a statistically significant correlation between epileptic spasms’ semiology and etiology: flexor spasms were associated with spasms due to a genetic cause; however, mixed spasms were associated with spasms from a structural cause. In 73% of patients with slow and sharp waves or slow waves on the ictal EEG, and asymmetric hypsarrhythmia or hemi hypsarrhythmia on the interictal EEG, we found spasms with structural etiology. However, 69% of patients with genetic etiology presented typical interictal hypsarrhythmia with high-amplitude polymorphic delta with multifocal spike or modified hypsarrhythmia on interictal EEG and slow waves on the ictal EEG.

### 6.1. Study limitations

The sample of the study should be larger, the reporting of the EEG should always be performed by the same operator as well as the clinical features should not be collected retrospectively, as there is a greater risk of error, and should always be described by an operator. The incidence of the causes of IS that we described does not fully reflect what is generally found in clinical practice and this could bias the results we have found. Finally, the standardization of the electroencephalographic characteristics should be carried out.

## Data availability statement

The original contributions presented in the study are included in the article/supplementary material, further inquiries can be directed to the corresponding author.

## Ethics statement

The studies involving human participants were reviewed and approved by the ethics committee of the University Hospital of Catania University and the Hospital of Buenos Aires. Written informed consent to participate in this study was provided by the participants’ legal guardian/next of kin.

## Author contributions

RF and RC contributed substantially to the conception and design of the work. LT and SS organized the database. GP performed the statistical analysis. GC, ADN, and SS wrote the first draft of the manuscript. All authors contributed to the review of the manuscript, read and approved the submitted version.

## Conflict of interest

The authors declare that the research was conducted in the absence of any commercial or financial relationships that could be construed as a potential conflict of interest.

## Publisher’s note

All claims expressed in this article are solely those of the authors and do not necessarily represent those of their affiliated organizations, or those of the publisher, the editors and the reviewers. Any product that may be evaluated in this article, or claim that may be made by its manufacturer, is not guaranteed or endorsed by the publisher.

## Abbreviations

ES: Epileptic spasms
EEG: Electroencephalogram
EMG: Electromyography
HIE: Hypoxic–Ischemic Encephalopathy
IS: Infantile Spasms
HMEG: Hemimegalencephaly
OR: Odds Ratio
PEHO: Progressive encephalopathy with Edema, Hypsarrhythmia, and Optic atrophy
TORCH: Toxoplasmosis, Others, Rubeola, Cytomegalovirus, Herpes
